# Phenotypic Variability in Siblings With Autosomal Recessive Polycystic Kidney Disease

**DOI:** 10.1016/j.ekir.2022.04.095

**Published:** 2022-05-04

**Authors:** Ramona Ajiri, Kathrin Burgmaier, Nurver Akinci, Ilse Broekaert, Anja Büscher, Ismail Dursun, Ali Duzova, Loai Akram Eid, Marc Fila, Michaela Gessner, Ibrahim Gokce, Laura Massella, Antonio Mastrangelo, Monika Miklaszewska, Larisa Prikhodina, Bruno Ranchin, Nadejda Ranguelov, Rina Rus, Lale Sever, Julia Thumfart, Lutz Thorsten Weber, Elke Wühl, Alev Yilmaz, Jörg Dötsch, Franz Schaefer, Max Christoph Liebau

**Affiliations:** 1 Department of Pediatrics, University Hospital Cologne and Faculty of Medicine, University of Cologne, Cologne, Germany; 2 Department of Pediatric Nephrology, Şişli Etfal Training and Research Hospital, İstanbul, Turkey; 3 Department of Pediatrics II, University Hospital Essen, Essen, Germany; 4 Department of Pediatric Nephrology, Erciyes University, Faculty of Medicine, Kayseri, Turkey; 5 Division of Pediatric Nephrology, Department of Pediatrics, Hacettepe University Faculty of Medicine, Ankara, Turkey; 6 Department of Pediatric Nephrology, Dubai Kidney Center of Excellence, Dubai Hospital, Dubai, United Arab Emirates; 7 Pediatric Nephrology Unit, CHU Arnaud de Villeneuve-Université de Montpellier, Montpellier, France; 8 Department of General Pediatrics and Hematology/Oncology, Children’s University Hospital Tuebingen, Tuebingen, Germany; 9 Division of Pediatric Nephrology, Research and Training Hospital, Marmara University, Istanbul, Turkey; 10 Division of Nephrology, Department of Pediatric Subspecialties, Bambino Gesù Children’s Hospital—IRCCS, Rome, Italy; 11 Pediatric Nephrology, Dialysis and Transplant Unit, Fondazione IRCCS Cà Granda, Ospedale Maggiore Policlinico, Milan, Italy; 12 Department of Pediatric Nephrology and Hypertension, Faculty of Medicine, Jagiellonian University Medical College, Krakow, Poland; 13 Department of Inherited and Acquired Kidney Diseases, Veltishev Research and Clinical Institute for Pediatrics of the Pirogov Russian National Research Medical University, Moscow, Russia; 14 Pediatric Nephrology Unit, Hôpital Femme Mère Enfant, Hospices Civils de Lyon, Centre de référence maladies rénales rares, Bron, France; 15 Department of Pediatrics, Saint-Luc Academic Hospital, Université Catholique de Louvain Medical School, Brussels, Belgium; 16 Division of Nephrology, University Children’s Hospital Ljubljana, Ljubljana, Slovenia; 17 Department of Pediatric Nephrology, Cerrahpaşa School of Medicine, Istanbul University Cerrahpasa, Istanbul, Turkey; 18 Department of Pediatric Gastroenterology, Nephrology and Metabolic Diseases, Charité—Universitätsmedizin Berlin, Berlin, Germany; 19 Division of Pediatric Nephrology, Center for Pediatrics and Adolescent Medicine, University of Heidelberg, Heidelberg, Germany; 20 Pediatric Nephrology Department, Istanbul University Istanbul Medical Faculty, Istanbul, Turkey; 21 Division of Pediatric Nephrology, Heidelberg University Center for Pediatrics and Adolescent Medicine, Heidelberg, Germany; 22 Center for Molecular Medicine, University Hospital Cologne and Faculty of Medicine, University of Cologne, Cologne, Germany

**Keywords:** ARPKD, Ciliopathies, *DZIP1L*, Fibrocystin, PKD, *PKHD1*

## Abstract

**Introduction:**

Autosomal recessive polycystic kidney disease (ARPKD) is a rare monogenic disorder characterized by early onset fibrocystic hepatorenal changes. Previous reports have documented pronounced phenotypic variability even among siblings in terms of patient survival. The underlying causes for this clinical variability are incompletely understood.

**Methods:**

We present the longitudinal clinical courses of 35 sibling pairs included in the ARPKD registry study ARegPKD, encompassing data on primary manifestation, prenatal and perinatal findings, genetic testing, and family history, including kidney function, liver involvement, and radiological findings.

**Results:**

We identified 70 siblings from 35 families with a median age of 0.7 (interquartile range 0.1–6.0) years at initial diagnosis and a median follow-up time of 3.5 (0.2–6.2) years. Data on *PKHD1* variants were available for 37 patients from 21 families. There were 8 patients from 7 families who required kidney replacement therapy (KRT) during follow-up. For 44 patients from 26 families, antihypertensive therapy was documented. Furthermore, 37 patients from 24 families had signs of portal hypertension with 9 patients from 6 families having substantial hepatic complications. Interestingly, pronounced variability in the clinical course of functional kidney disease was documented in only 3 sibling pairs. In 17 of 20 families of our cohort of neonatal survivors, siblings had only minor differences of kidney function at a comparable age.

**Conclusion:**

In patients surviving the neonatal period, our longitudinal follow-up of 70 ARPKD siblings from 35 families revealed comparable clinical courses of kidney and liver diseases in most families. The data suggest a strong impact of the underlying genotype.

## Graphical abstract

**Figure:**
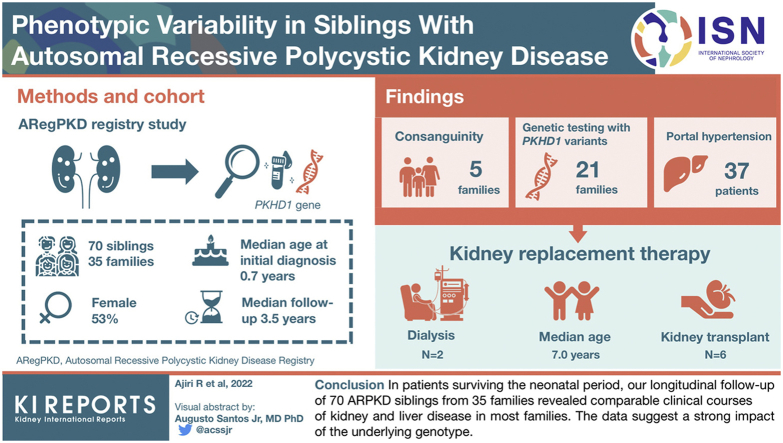

See Commentary on Page 1453

ARPKD is a severe genetic disorder that usually manifests clinically in early childhood and is characterized by fibrocystic changes in the kidneys and the liver.^1^ It is mainly caused by variants in the *PKHD1* gene on chromosome 6 encoding the protein fibrocystin.^2^^,^^3^ The estimated incidence of ARPKD is approximately 1:20,000 live births.^2^ Severely affected neonates may die from respiratory insufficiency because of pulmonary hypoplasia. Approximately 50% of the patients who survive the neonatal period develop chronic kidney failure within the first 2 decades of life, and ARPKD is one of the most common indications for KRT in children.^3^^,^^4^ Additional clinical manifestations include bile duct dilatation, periportal fibrosis, and portal hypertension as signs of hepatic involvement because of ductal plate malformation.^3^^,^^4^ A subset of patients may develop life-threatening complications arising from cholangitis or gastrointestinal bleeding secondary to portal hypertension.^5^^,^^6^ Although prenatal or neonatal presentations are most common, atypical late manifestations, even in adulthood, have been described.^1^

The molecular mechanisms underlying the clinical variability are poorly understood.^7^ Genotype-phenotype correlations have been limited to specific *PKHD1* constellations for a long time. Severe phenotypes, for example, with perinatal or neonatal demise were found in patients carrying 2 truncating *PKHD1* variants.^3^ It was discussed that at least 1 missense variant would be required for neonatal survival, even though also patients with 2 missense variants could have severe phenotypes.^8^ More recently, however, various patients with 2 truncating *PKHD1* variants surviving the neonatal period have been described and more precise insights into genotype-phenotype correlations were obtained in a large cohort of patients with longitudinal follow-up.^9^ Recent data suggest that the affected regions in *PKHD1* may also have an important influence on the phenotype in case of missense variants.^9^ A potential role of modifier genes in ARPKD has been suggested.^10^, ^11^, ^12^

Studying siblings with ARPKD may give important insights to understand mechanisms underlying phenotypical variability. Previous studies described clinical variability among siblings with ARPKD, but most of the work focused on patient survival as a readout.^12^, ^13^, ^14^, ^15^ As neonatal treatment options are improving, estimating long-term courses in ARPKD is becoming even more relevant. However, the literature on siblings with ARPKD is limited in quantity and clinical depth, including up-to-date genetic information. Here, we therefore analyze the phenotypic variability of 35 sibling pairs with ARPKD from the ARPKD registry study ARegPKD. To the best of our knowledge, this is the largest cohort with longitudinal follow-up data of many genotyped sibling pairs.

## Methods

### ARegPKD Registry Study

Patients were followed up in the ARegPKD registry study as previously described.^16^ In brief, ARegPKD is an international, multicenter, prospective and retrospective observational study. The inclusion criteria consist of the diagnosis of ARPKD by molecular findings, histology, or clinical findings according to the criteria of Zerres *et al.*^17^ Both, children and adult patients with the diagnosis of ARPKD are included. Patients with genetic, histologic, or clinical proof of another cystic kidney disease are excluded. The registry study was approved by local ethics committees and is in accordance with the ethical standards laid down in the Declaration of Helsinki in 1964 and its follow-ups. The registry gathers data during mostly annual visits; the time schedule is, however, flexible, allowing for different time intervals. Furthermore, data about primary manifestation, prenatal and perinatal findings, genetic testing, and family history are collected, including clinical, imaging, and laboratory variables as provided by the centers. Data entered into the web-based database are automatically checked using predefined plausibility analyses to avoid erroneous or incomplete data and were reviewed by a pediatric nephrologist.

*PKHD1* variants were classified in accordance with the revised criteria of the American College of Medical Genetics and additionally categorized by their probable impact on protein translation.^18^ Only variants of uncertain significance (VUS), likely pathogenic (LP), or pathogenic (P) variants according to the American College of Medical Genetics classification were reported. Genetic classification of patients according to diagnostic certainty into subgroups “confirmed,” “probable,” and “unknown” was done as previously described for patients with detected *PKHD1* variants (“confirmed” for patients with ≥2 *PKHD1* variants detected, with ≥2 classified as LP or P; “probable” for ≥2 *PKHD1* variants detected, with only 1 classified as LP or P; “unknown” for ≥2 *PKHD1* VUS detected, only 1 *PKHD1* variant detected, classified as a VUS, LP or P)^9^ and included “unknown” for patients without detected *PKHD1* variants.

Estimated glomerular filtration rates (eGFRs) were calculated according to the full age spectrum formula using the eGFR value at the last provided visit.^19^ Because the full age spectrum eGFR is not validated for children <1 year of age, their eGFRs were calculated according to the bedside Schwartz formula. Kidney Disease: Improving Global Outcomes classification was used to categorize kidney function into stages of chronic kidney disease (CKD).^20^ The start of KRT was defined by onset of any type of dialysis or the date of kidney transplantation or combined liver and kidney transplantation.

Liver disease was classified as previously described.^9^ Portal hypertension was defined by a first episode of documented thrombocytopenia (platelet count <150,000/μl) or presence of sonographic splenomegaly or collateral blood flow (varices) or a substantial hepatic complication. A substantial hepatic complication was defined as documentation of variceal bleeding, interventional or surgical generation of portosystemic shunts, or liver transplantation. Sonographic splenomegaly was defined according to the upper limits of normal as suggested in pediatric and adult reference studies. Splenomegaly was therefore defined as spleen length > mean + 2 SD in pediatric and ≥13.0 cm in adult patients.^21^, ^22^, ^23^

Renal sonographic results were obtained from local clinical routine imaging, were correlated to height, and were compared between siblings if data points were available within a time frame of <3 months in the first year of life, a time frame of <6 months between the first and the third year, and a difference of <12 months in children beyond 3 years of age (“comparable visit”). For comparison between siblings, the ratio of the mean height-adjusted values of both kidneys was calculated by dividing the height-adjusted pole-to-pole length of the older sibling by the height-adjusted pole-to-pole length of the younger sibling.

### Statistical Analysis

Statistical analysis was conducted using IBM SPSS Statistics 26 (Chicago, IL). This study mainly focuses on descriptive analyses. Data analysis was performed on a data set available by March 2020, and data of siblings with ARPKD were filtered. Continuous variables were expressed as median (interquartile range). Counts and proportions were expressed as number/total number of informative cases (percentage). Informative cases were defined as cases with sufficient information for a specific analysis. Numbers therefore vary according to data availability.

## Results

### Cohort Description

By March 2020, data of 624 patients were available in the ARegPKD registry. For 374 of these patients, a sibling has been documented. Of those patients with a documented sibling, 35 pairs of siblings with ARPKD from 20 different participating centers from 9 different countries were registered. One additional sibling was left with the information of having ARPKD, but was not listed in our registry, and 1 sibling died from lung hypoplasia postnatally without additional available information and could thus not be included in the analysis (Supplementary Figure S1). The 35 pairs of ARPKD siblings include 3 pairs of twins. A total of 356 visits were available for the description of clinical courses.

**Figure 1:**
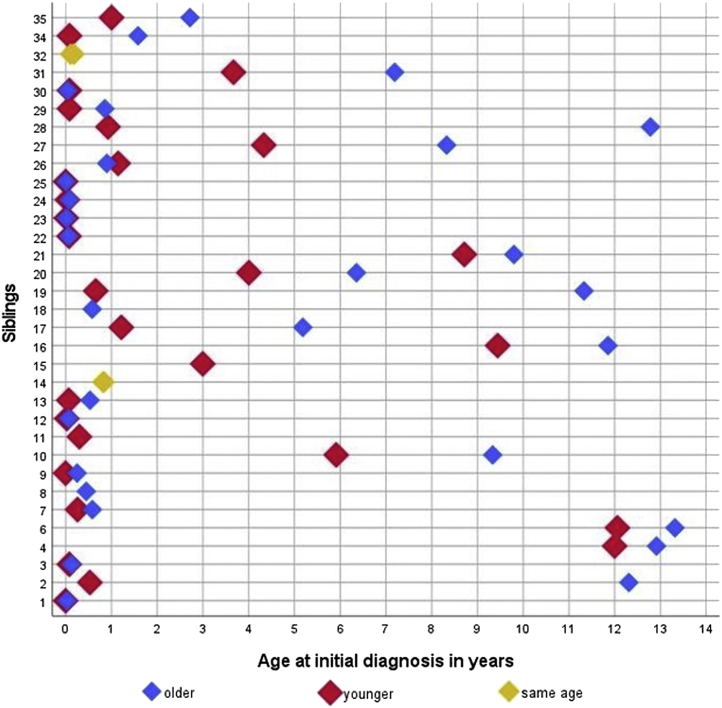
Siblings’ ages at initial diagnosis of ARPKD. Note that in families 14 and 32, both siblings are represented by 1 symbol. Data were not available for families 5 and 33 and 1 sibling each in families 8, 11, 15, and 18. ARPKD, autosomal recessive polycystic kidney disease.

Most children were diagnosed during the first year of life (33 of 62; 53% of informative cases; Table 1). The initial diagnosis of ARPKD was made either simultaneously in both siblings or at a younger age in the younger sibling except for sibling pair 26, in which the older sibling was diagnosed at a younger age (Figure 1 and Supplementary Table S1). In most cases with available information, the older sibling was the index patient of the family (11 of 16; 69%). Detailed patient characteristics are presented in Table 1 and Supplementary Table S1. One patient died in the neonatal period at the age of 25 days because of respiratory failure (family 1, older sibling).

**Table 1:** Patient characteristics of 70 patients from 35 sibling pairs with ARPKD

Patient characteristics	Percentage (%) or median (IQR) of *N* informative cases
General characteristics	
Sex (female, male %)	53, 47
Age at initial diagnosis (*n =* 62), yr	0.7 (0.1–6.0)
- Subgroups (%)	
Prenatal *n/N* (%)	3/62 (4.8)
0–1 yr *n/N* (%)	33/62 (53)
1–5 yr *n/N* (%)	9/62 (15)
5–10 yr *n/N* (%)	9/62 (15)
≥10 yr *n/N* (%)	8/62 (13)
- older sibling, yr	0.9 (0.1–9.5)
- younger sibling, yr	0.5 (0.1–3.3)
Year of initial diagnosis (*n =* 62), median (IQR)	2012 (2009–2015)
Age at last visit (n = 67), yr	9.0 (5.0–16.0)
Number of visits (*n =* 356)	7.0 (5.0–12.0)
Time of follow-up, yr	3.5 (0.2–6.2)
*PKHD1* genetic sequencing performed, *n/N* (%)	39/65 (60.0)
- ≥1 *PKHD1* variant detected (VUS, LP, P), *n/N* (%)	37/39 (94.8)
- ≥2 *PKHD1* variant detected (VUS, LP, P), *n/N* (%)	34/39 (87.2)
- Genetic status: confirmed	26/39 (66.7)
- Genetic status: probable	0/39 (0)
- Genetic status: unknown	13/39 (33.3)
Perinatal findings	
Prenatal oligohydramnios or anhydramnios, *n/N* (%)	13/57 (22.8)
Prenatal increased renal echogenicity, *n/N* (%)	12/57 (21.1)
Gestational age at birth (*n =* 40), wk	38.0 (37.0–39.0)
Birth weight (*n =* 46), g	3020 (2695–3338)
APGAR 10 min (*n =* 27)	9 (8–10)
Admission to NICU, *n/N* (%)	13/62 (21.0)
Days on NICU (*n =* 13)	15.0 (4.5–26.0)
Assisted breathing and/or ventilation, *n/N* (%)	9/61 (14.8)
Kidney events	
KRT, *n/N* (%)	8/67 (11.9)
Age at start of KRT (*n =* 8), yr	7.0 (3.8–10.5)
Liver events	
Signs of portal hypertension, *n/N* (%)	37/67 (55.2)
- Age at initial diagnosis (*n =* 37), yr	6.0 (3.5–13.5)
- Splenomegaly, *n/N* (%)	32/53 (60.4)
- Thrombocytopenia, *n/N* (%)	25/64 (39.1)
- Varices, *n/N* (%)	14/65 (21.5)
Substantial hepatic complications, *n/N* (%)	9/67 (13.4)
- Age at initial diagnosis (*n =* 9), yr	8.0 (4.5–12.0)
Cardiovascular events	
Use of antihypertensive medication, *n/N* (%)	44/67 (65.7)
Signs of left ventricular hypertrophy, *n/N* (%)	7/24 (29.2)

ARPKD, autosomal recessive polycystic kidney disease; IQR, interquartile range; KRT, kidney replacement therapy; LP, likely pathogenic (class 4); NICU, neonatal intensive care unit; P, pathogenic (class 5); VUS, variant of uncertain significance (class 3).

### Parents and Family History

Consanguinity was documented for 5 families; in 26 families, the parents were not consanguineous; and in 4 families, the status was unknown (Supplementary Table S1). One miscarriage each was reported in 3 families.

The median (interquartile range) age of the father at birth of the older sibling and the twins was 29.5 years (*n =* 23; 26.3–35.0), and the median age of the mother was 27.0 years (*n =* 23; 20.0–33.0). The median age of the father at birth of the younger sibling was 34.0 years (*n =* 20; 28.3–39.5), and the median age of the mother was 30.5 years (*n =* 20; 27.5–35.8).

### Genetic Findings

Details of genetic sequencing of *PKHD1* are presented in Supplementary Table S1. *PKHD1* analysis was performed in 39 (39 of 65, 60%) patients from 22 different families. Both siblings had documented genetic analysis in 18 families, with only 1 child in 4 families. Variants were detected in 37 (95%) patients from 21 different families with an allele detection rate of 92% in the tested patients (72 of 78). There were 34 patients who carried at least 2 *PKHD1* variants. Furthermore, 26 patients from 14 of 22 tested families were genetically confirmed with 2 P or LP *PKHD1* variants (64% of families with genetic testing, 40% of the overall cohort). Thus, 21 of the overall 35 families were not genetically confirmed (60% of families of the overall cohort) because there was either no *PKHD1* analysis performed or no documentation of 2 P or LP *PKHD1* variants. Of these 21 families without genetic confirmation, 8 underwent *PKHD1* analysis with patients from 4 of these families carrying 2 (families 8, 28, and 35) or 3 (family 2) *PKHD1* VUS and 1 patient each from an additional 3 families (families 24, 26, and 29) with detection of a single P *PKHD1* variant. One family (family 15) remained without detection of a *PKHD1* variant. In 1 family, a variant of *PKHD1* was detected in the older sibling and was not detected in the younger one (family 24). For genetic workup, further polycystic kidney disease genes were analyzed, *PKD1* and *PKD2* in 12, *HNF1B* in 8, and *DZIP1L* and *GANAB* in 5 children each without detection of variants.

### Clinical Course

#### Perinatal Respiratory Disease

Overall, there was mild perinatal respiratory disease in the evaluated cohort with limited need for treatment on neonatal intensive care unit. Only 3 (4%) children received perinatal mechanical ventilation, including both siblings of sibling pair 1. Both children were born prematurely, one at 31 + 1 weeks of gestational age with a birth weight of 2010 g and the other, who died early because of respiratory failure, at 34 + 6 weeks of gestational age with a birth weight of 2600 g. For general perinatal findings of the cohort; see Table 1 and Supplementary Table S2.

#### Kidney Disease

For 66 patients, data on eGFR at their last documented visit were available. CKD G stages are presented in Supplementary Table S1.

**Figure 2:**
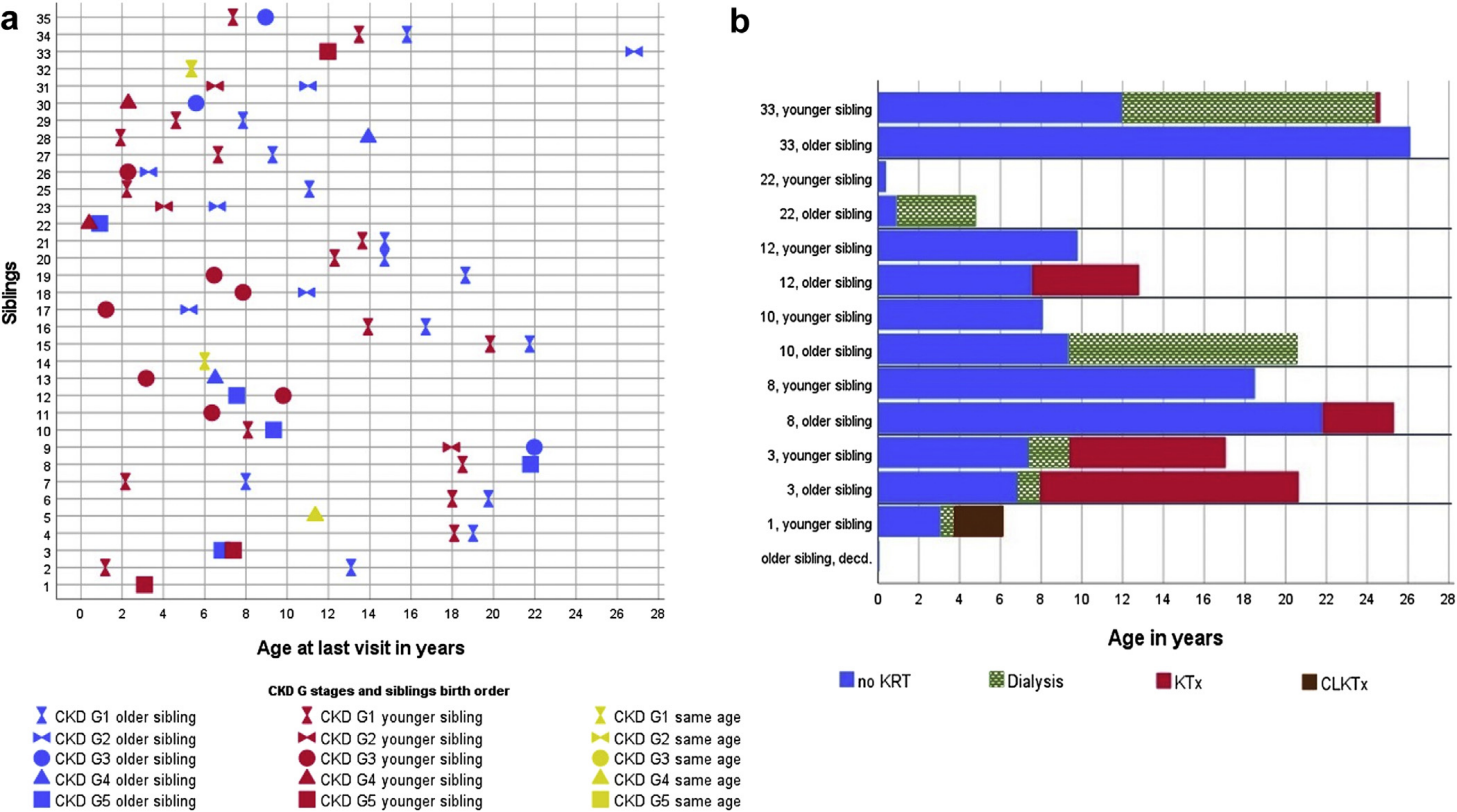
(a) Depiction of different CKD stages of native kidney function in sibling pairs. Families 5, 14, and 32 are depicted by 1 symbol. For patients or families not represented, clinical data were not available. (b) Course of patients requiring KRT and their sibling pair. CKD, chronic kidney disease; CLKTx, combined liver and kidney transplantation; decd., deceased; KRT, kidney replacement therapy; KTx, kidney transplantation.

The median age difference between siblings at the last available follow-up visit with native kidney function was 3 (1.0–4.3) years. There were 18 (56%) pairs of siblings who were in the same CKD G stage at the last documented visit. Furthermore, 14 (44%) pairs of siblings were in different CKD G stages at that time point. Half of these pairs had a difference of just a single CKD G stage, 3 pairs had a difference of 2 CKD G stages (families 12, 19, and 35), 2 sibling pairs had a difference of 3 CKD G stages (families 28 and 33), and 2 additional pairs had a difference of 4 stages (families 8 and 10). In 3 families, the age difference for a last visit with native kidney function was ≥12 years. These families had a difference of 2 CKD G stages in family 19 and 3 CKD G stages in families 28 and 33 (Figure 2a and Supplementary Table S1). As expected, lower CKD G stages were more frequently found in the younger siblings with documentation at younger ages.

Comparable data for the CKD G stages at similar ages were given in 20 families (20 of 35; 57%) in 156 visits with a median age of 5.8 years (2.5–11.4). The CKD G stages were defined as comparable when there was a maximum age difference of 3 months between the siblings at the age of 0 to 1 years, a difference of 6 months at the age of 1 to 3 years, and a difference of 1 year at the age of 3 years and older. We looked more in detail into the last comparable visits (*n =* 40) between the sibling pairs with a median age of 10.6 years (4.3–16.6). Detailed information is depicted in Supplementary Table S1.

At these visits, 12 sibling pairs (60%) were in the same CKD G stage at a median age of 12.6 years (6.1–16.9). There were 5 sibling pairs (25%) who had a difference of 1 CKD G stage at a median age of 4.0 years (3.3–10.4), and 2 families had a difference of 2 CKD G stages (families 12 and 30). In sibling pair 12, the younger sibling was in CKD stage G3 and the older one in CKD stage G5. In sibling pair 30, the older sibling was in CKD stage G2 and the younger one in CKD stage G4. Family 33 had a difference of 3 CKD G stages (CKD G5 in the younger sibling vs. CKD G2 in the older sibling).

KRT was initiated in 8 (11%) patients at a median age of 7.0 (3.8–10.5) years (Table 1, Figure 2b). At the time of the investigation, 6 patients (9%) were transplanted (either kidney transplantation or combined liver and kidney transplantation) and 2 patients (3%) were on dialysis (family 10, older sibling; family 22, older sibling).

There was no substantial difference between the patients who needed KRT and their siblings without KRT concerning perinatal gestational age, birth weight, and perinatal APGAR scores, although available data remained limited (Supplementary Table S2; for general information, see Table 1).

To compare kidney sizes between siblings, 164 visits from 30 families with height-adjusted pole-to-pole-lengths of the left and right kidneys were evaluated. The ratio of the height-adjusted mean values of both kidneys (average height-adjusted pole-to-pole lengths) was calculated and compared in 14 families based on 64 visits with overall minor differences between the siblings (Supplementary Table S1). Differences were observed in families 5, 33, and 34.

#### Hypertension, Cardiovascular Disease, and Hepatic Disease

Numbers of documented cardiovascular and hepatic events are presented in Table 1 and Supplementary Table S1. Defined variability was observed for cardiovascular and hepatic diseases and corresponding treatment.

In 18 families, both siblings were treated antihypertensively; in 7 families, only 1 patient received antihypertensive medication (3 times the older sibling, 4 times the younger sibling). In 1 family, only 1 of the twins was treated with antihypertensive medication. Furthermore, 7 patients from 5 different families had signs of left ventricular hypertrophy. In 1 patient, dilatation of the left heart chamber and minimal mitral and aortic insufficiency were described.

**Figure 3:**
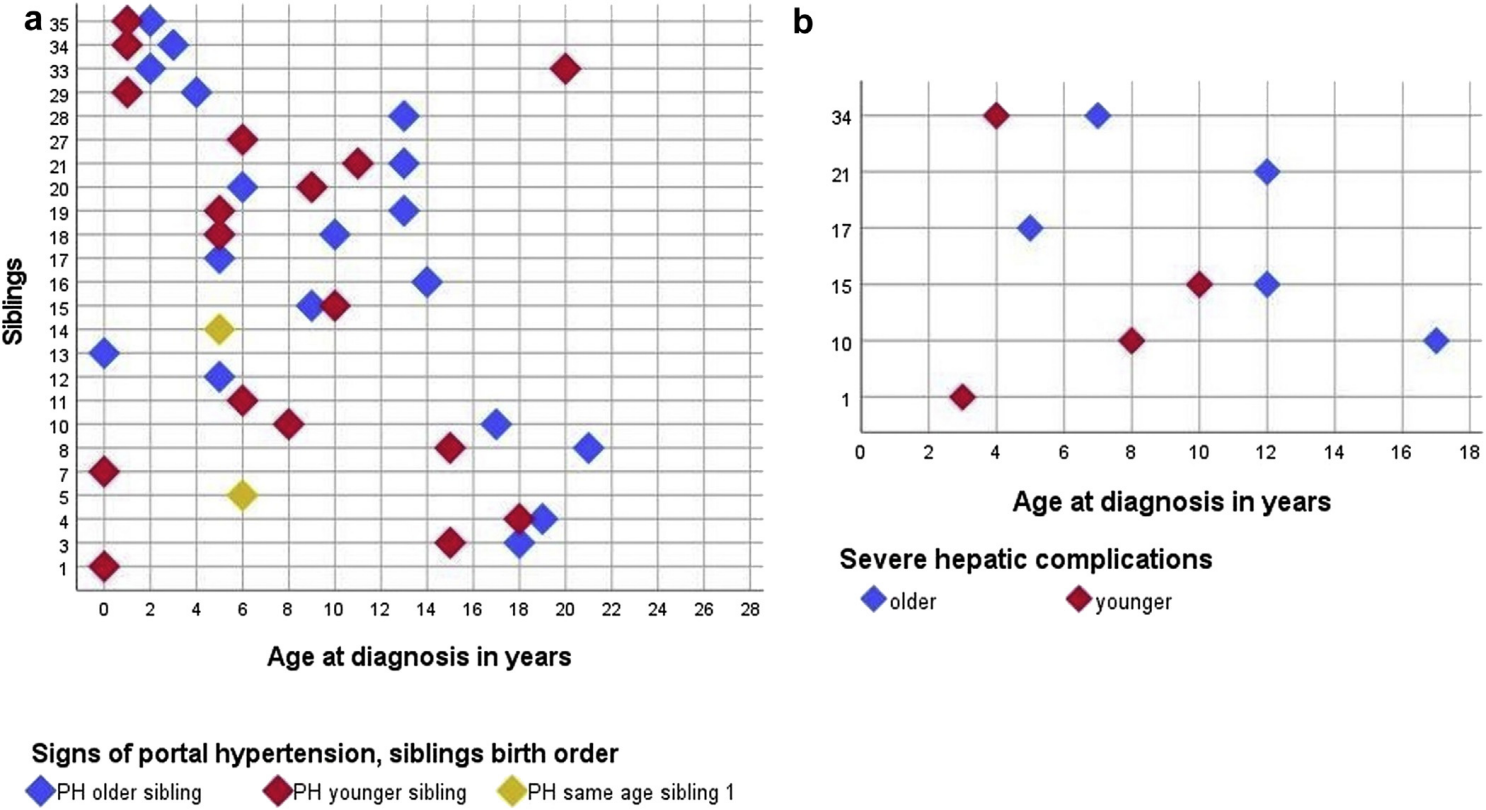
Age at (a) first available documentation of signs of PH and (b) severe hepatic complications. The patients without documented signs of PH or severe hepatic complications are not depicted. Family 1 older sibling is not depicted because of early death. PH, portal hypertension.

Thirty seven patients from 24 different families had signs of portal hypertension (Table 1, Figure 3a). In 9 families, both siblings had splenomegaly; in 14 families, only 1 sibling had splenomegaly. In 6 families, thrombocytopenia was documented in both siblings; in 13 families, only 1 sibling had documentation of thrombocytopenia. In 4 families, both siblings were affected by esophageal or gastric varices; in 6 families, only 1 child was affected. Median age of initial diagnosis in families with both patients having signs of portal hypertension was 2.7 (0.5–9.0) years. Signs of portal hypertension were overall more often found in the older siblings.

Furthermore, 3 pairs of siblings (families 10, 15, and 34) and 3 patients from 3 different families (families 1, 17, and 21) had substantial hepatic complications (Figure 3b). In the 3 latter families, there was limited follow-up in families 1 (early death of older sibling) and 17 (last visit of younger sibling at age 1.2 years). In family 21, the sibling without substantial hepatic complications had signs of portal hypertension. Only 1 patient received a combined liver and kidney transplantation (family 1, younger patient); 4 patients had variceal bleeding including 1 pair of siblings and 2 siblings from different families (the older sibling each). There were 5 patients from 3 families (families 15, 17, and 34) who underwent either a sclerosing or banding of varices (in family 17, only the older sibling). Median age of initial diagnosis in families with both siblings having severe hepatic complications was 3.0 (0.8–7.6) years.

Signs of hepatic involvement were overall more frequently detected at a younger age in the younger siblings (median age of the younger sibling at documentation of signs of liver involvement was 7.0 years, and median age of the older sibling was 12.0 years).

#### Pronounced Clinical Differences Between Siblings

There were 3 sibling pairs that had pronounced differences during the course of their disease (Supplementary Table S1).

In sibling pair 8, the older sibling was first classified as CKD stage G5 at 21 years. Signs of portal hypertension were first documented at the age of 21 years. In case of the younger sibling, the patient was classified as CKD stage G1 at the age of 18.5 years. Signs of portal hypertension were first documented at the age of 15 years. Genetic testing revealed 2 *PKHD1* missense variants (both classified as VUS) in both siblings (c.4738C>T; C.4913T>C).

In sibling pair 10, the older sibling was classified as CKD stage G5 at the age of 9 years. The patient had signs of severe hepatic complications at the age of 17 years. The younger sibling was classified as CKD stage G1 and had signs of severe hepatic complications both at the age of 8 years. Genetic testing revealed 2 *PKHD1* missense variants (classes LP and P in both siblings [c. 5426G>A; C. 10444C>T)]).

In sibling pair 33, the older patient was classified as CKD stage G2 at the age of 26.8 years and had signs of portal hypertension at the age of 2 years. The younger sibling was classified as CKD stage G5 at the age of 11.9 years but signs of portal hypertension were first documented at the age of 20 years. As mentioned previously, there was a difference in kidney size between the siblings with the younger, more severely affected patient, having larger kidneys.

Less pronounced but clearly detectable differences were identified in additional families including differences in kidney size in families 5 and 34 (Supplementary Table S1), hepatic involvement (see above), and CKD G stages*.* A difference of 2 CKD G stages at the last comparable visit was found in families 12 and 30. A difference of 2 CKD G stages was found at the visits with an age difference of 1.5 years in family 35. In family 19, the younger sibling was in CKD G 3 at the age of 6.5 years and the older one in CKD G 1 at the age of 18.6 years.

## Discussion

In this manuscript, we present data on disease courses of siblings having ARPKD. The clinical heterogeneity observed in ARPKD remains only partly understood. In addition to the type of variant, the affected region in the *PKHD1* gene seems to be relevant for determination of clinical courses in patients with missense variants.^9^

A number of case reports have revealed phenotypic variability between ARPKD sibling pairs.^14^^,^^15^^,^^24^^,^^25^ A long-term prospective and retrospective multicenter study from 1995 compared the phenotypic variability of 20 siblings with ARPKD.^13^ These siblings were diagnosed with having ARPKD according to typical clinical signs defined by Cole^26^ and Zerres*.*^27^ Genetic details were not available in these studies. In a study describing 186 patients with analyses of *PKHD1* genotypes, Bergmann *et al.*^12^ presented marked intrafamilial phenotypic variability in 20 of 48 (42%) sibships with demise of one of the siblings in the neonatal period (22 neonatal demises in 20 families). The variability of clinical courses and the severity of the disease in these studies were mainly defined by neonatal survival.

Multiple aspects in our understanding and clinical management of ARPKD have substantially changed since the mentioned important studies were performed. Neonatal intensive care treatment has greatly developed in recent years, and more children with ARPKD can survive the neonatal period and reach childhood or even adulthood. Yet, data on longitudinal clinical courses in genetically characterized ARPKD siblings are scarce. Furthermore, genetic diagnostic tools have massively improved during the recent years and have revealed that the genetic basis for early onset cystic kidney diseases is complex. It is nowadays also well known that variants in *PKHD1* are the primary cause of ARPKD but that the phenotype can also be mimicked by variants in other genes. The presence of multiple allelism as a reason for phenotypic variability has been discussed for ARPKD.^11^^,^^13^ The role of specific modifier genes in ARPKD remains to be investigated in detail. For autosomal dominant polycystic kidney disease, preclinical data have revealed that the genetic background may influence disease severity.^28^

In this study, we compared 35 pairs of siblings affected by ARPKD deriving from the ARegPKD database, thus allowing a longitudinal study of clinical phenotypes. Only 1 patient died in the neonatal period. It has previously been found that neonatal survival is a crucial landmark in ARPKD. Children surviving the first weeks of life had high chances of survival in the following years. The focus of previous studies on survival as a marker of clinical variability may have strongly influenced the perception of clinical variability. Our cohort did not reveal pronounced perinatal disease with only 21% of patients being admitted to neonatal intensive care and 15% of patients requiring respiratory support. This observation has various implications. On one hand, good perinatal courses may have influenced the subsequent clinical developments, for example, of CKD. Ventilation has repetitively been found to be associated with poorer kidney outcome in ARPKD.^4^^,^^7^ On the other hand, it is tempting to speculate that the experiences of mild respiratory disease in the first child may have influenced the way parents feel and decide about having a second child with ARPKD.

For >60% of the families included in this study, genetic testing with detection of *PKHD1* variants was documented in at least 1 patient. Only a few of the patients were classified as genetically confirmed with detection of 2 P or LP *PKHD1* variants. We have previously revealed that there is no clear correlation between genetic confirmation status and clinical course of kidney and liver diseases in patients with at least 1 *PKHD1* variant classified as VUS, LP, or P, including patients with only a single detected variant.^9^

Among all families, only 3 sibling pairs had substantial variability in the course of kidney disease (families 8, 10, and 33) with 1 sibling having KRT and the other only having mild affection of the kidney disease. Of these families, 2 had biallelic *PKHD1* missense variants. Both families were from Algeria. The identified *PKHD1* variants differed between the 2 families. There was no documented consanguinity in these families. Families 10 and 33 also had differing courses of liver disease. Interestingly, 4 of the 6 patients from these 3 families were born in the 1990s, which may point to a more uniform treatment in more recently born children. In our cohort, 2 additional families had differences in kidney function at comparable ages (families 12 and 30) without a need of KRT. All other sibling pairs had limited variability in their kidney or liver disease courses.

Our study has some important implications for counseling of families. The major strength of this study is the multinational approach covering a large number of genotyped siblings with ARPKD and recent clinical data (Supplementary Figure S2). Our data included families with very good longitudinal follow-up for both concordant and discordant courses. Although we detected some degree of variability, we observed more clinical consistency in the course of kidney and liver diseases among siblings than anticipated. These data suggest a strong influence of the underlying genotype for clinical courses in childhood and adolescence and may be a relevant information for affected families expecting a second child. Given the low number of sibling pairs with diverging clinical courses, we did not initiate an analysis for potential clinical risk factors affecting the disease course of siblings. Interestingly, we did not detect direct evidence for better disease courses of younger siblings as a potential effect of earlier diagnosis and concomitant initiation of treatment. Numbers may have been too small and the follow-up time too short to identify such effects. Earlier disease detection and closer medical follow-up in younger siblings may have resulted in a more precise documentation of early stages of CKD and liver involvement, for example, documentation of thrombocytopenia.

There are several limitations to this descriptive study: as a registry study, there is a certain degree of selection bias because of the limited availability of data, especially for the most severe cases with perinatal death. Furthermore, follow-up time and clinical, genetic, and laboratory workup differ between patients and centers. Not all longitudinal courses were completely recorded, and some variability during the course of the disease may thus not have been captured by our data. In 2 children, no *PKHD1* variant was detected, although a P variant was detected in 1 of the affected siblings in 1 of these families. As mainly pediatric nephrology centers contribute to the ARegPKD registry, there may additionally be an underrepresentation of the hepatic phenotype of ARPKD. Furthermore, the ARegPKD consortium involves mainly tertiary care centers, which may lead to a bias in the way that patients with milder phenotypes not requiring KRT were not included in our registry. As a further limitation, potential modifier genes or genetic variants in other PKD genes were not systematically analyzed and numbers remain too small to study effects of variant types.

In summary, our study provides new descriptive insights into clinical courses of siblings with ARPKD based on a large number of patients who survived the perinatal period and from many families with available information on *PKHD1* variants. The data suggest substantial consistency in the clinical courses of ARPKD siblings, which may be helpful for clinical counseling and can serve as a starting point for additional studies in independent cohorts.

## Disclosure

MCL has received honoraria for scientific lectures from Pfizer. Representing the University Hospital of Cologne, MCL has been counseling Otsuka in an advisory board. All the other authors declared no competing interests.

## Supplementary Material

Supplementary File (PDF)

**Figure S1.** Flowchart of data inclusion.

**Figure S2.** First documented visit before and after 2005.

**Table S1.** Overview of genetic testing and findings in PKHD1 in 70 patients from 35 sibling pairs with ARPKD. ARPKD, autosomal recessive polycystic kidney disease.

**Table S2.** Perinatal data of patients requiring KRT and their corresponding siblings. KRT, kidney replacement therapy.

**STROBE Statement.**

## References

[1] Burgmaier K., Kilian S., Bammens B. (2019). Clinical courses and complications of young adults with autosomal recessive polycystic kidney disease (ARPKD). Sci Rep.

[2] Zerres K., Mücher G., Becker J. (1998). Prenatal diagnosis of autosomal recessive polycystic kidney disease (ARPKD): molecular genetics, clinical experience, and fetal morphology. Am J Med Genet.

[3] Bergmann C., Senderek J., Sedlacek B. (2003). Spectrum of mutations in the gene for autosomal recessive polycystic kidney disease (ARPKD/PKHD1). J Am Soc Nephrol.

[4] Guay-Woodford L.M., Desmond R.A. (2003). Autosomal recessive polycystic kidney disease: the clinical experience in North America. Pediatrics.

[5] Davis I.D., Ho M., Hupertz V., Avner E.D. (2003). Survival of childhood polycystic kidney disease following renal transplantation: the impact of advanced hepatobiliary disease. Pediatr Transplant.

[6] Khan K., Schwarzenberg S.J., Sharp H.L. (2002). Morbidity from congenital hepatic fibrosis after renal transplantation for autosomal recessive polycystic kidney disease. Am J Transplant.

[7] Burgmaier K., Kunzmann K., Ariceta G. (2018). Risk factors for early dialysis dependency in autosomal recessive polycystic kidney disease. J Pediatr.

[8] Bergmann C. (2015). ARPKD and early manifestations of ADPKD: the original polycystic kidney disease and phenocopies. Pediatr Nephrol Berl Ger.

[9] Burgmaier K., Brinker L., Erger F. (2021). Refining genotype-phenotype correlations in 304 patients with autosomal recessive polycystic kidney disease and PKHD1 gene variants. Kidney Int.

[10] Hildebrandt F., Benzing T., Katsanis N. (2011). Ciliopathies. N Engl J Med.

[11] Bergmann C., Guay-Woodford L.M., Harris P.C. (2018). Polycystic kidney disease. Nat Rev Dis Primers.

[12] Bergmann C., Senderek J., Windelen E. (2005). Clinical consequences of PKHD1 mutations in 164 patients with autosomal-recessive polycystic kidney disease (ARPKD). Kidney Int.

[13] Deget F., Rudnik-Schöneborn S., Zerres K. (1995). Course of autosomal recessive polycystic kidney disease (ARPKD) in siblings: a clinical comparison of 20 sibships. Clin Genet.

[14] Barth R.A., Guillot A.P., Capeless E.L., Clemmons J.J. (1992). Prenatal diagnosis of autosomal recessive polycystic kidney disease: variable outcome within one family. Am J Obstet Gynecol.

[15] Kaplan B.S., Kaplan P., de Chadarevian J.P. (1988). Variable expression of autosomal recessive polycystic kidney disease and congenital hepatic fibrosis within a family. Am J Med Genet.

[16] Ebner K., Feldkoetter M., Ariceta G. (2015). Rationale, design and objectives of ARegPKD, a European ARPKD registry study. BMC Nephrol.

[17] Zerres K., Rudnik-Schöneborn S., Deget F. (1996). Autosomal recessive polycystic kidney disease in 115 children: clinical presentation, course and influence of gender. Acta Paediatrica.

[18] Richards S., Aziz N., Bale S. (2015). Standards and guidelines for the interpretation of sequence variants: a joint consensus recommendation of the American College of Medical Genetics and Genomics and the Association for Molecular Pathology. Genet Med.

[19] Pottel H., Hoste L., Dubourg L. (2016). An estimated glomerular filtration rate equation for the full age spectrum. Nephrol Dial Transplant.

[20] (2013). Kidney Disease: Improving Global Outcomes (KDIGO) CKD work group KDIGO 2012 clinical practice guideline for the evaluation and management of chronic kidney disease. Kidney Int Suppl.

[21] Rosenberg H.K., Markowitz R.I., Kolberg H. (1991). Normal splenic size in infants and children: sonographic measurements. AJR Am J Roentgenol.

[22] El Sharkawy E., Faris R., Grumbach K. (1997). Ultra sonographic measurements of the normal liver and spleen among Egyptians 10–50 years old. J Egypt Public Health Assoc.

[23] Yazdanpanah Y., Thomas A.K., Kardorff R. (1997). Organometric investigations of the spleen and liver by ultrasound in Schistosoma mansoni endemic and nonendemic villages in Senegal. Am J Trop Med Hyg.

[24] Gang D.L., Herrin J.T. (1986). Infantile polycystic disease of the liver and kidneys. Clin Nephrol.

[25] Kääriäinen H. (1987). Polycystic kidney disease in children: a genetic and epidemiological study of 82 Finnish patients. J Med Genet.

[26] Cole B.R., Gardner K.D., Bernstein J. (1990). The Cystic Kidney. Developments in Nephrology.

[27] Zerres K. (1992). Autosomal recessive polycystic kidney disease. Clin Investig.

[28] Arroyo J., Escobar-Zarate D., Wells H.H. (2021). The genetic background significantly impacts the severity of kidney cystic disease in the Pkd1RC/RC mouse model of autosomal dominant polycystic kidney disease. Kidney Int.

