# Supplementary material for: Phenotypic Variability in Siblings With Autosomal Recessive Polycystic Kidney Disease
Source: Kidney Int Rep. 2022 May 4;7(7):1643–52. doi: 10.1016/j.ekir.2022.04.095 (PMC9263410; doi:10.1016/j.ekir.2022.04.095)
Supplement: Supplementary File (PDF) [file mmc1.pdf]

Supplementary data of the manuscript

Phenotypic Variability in Siblings with Autosomal Recessive Polycystic Kidney Disease

Ramona Ajiri MD [1*], Kathrin Burgmaier MD [1*], Nurver Akinci MD [2], Ilse Broekaert MD [1], Anja Büscher MD [3], Ismail Dursun MD [4], Ali Duzova MD [5], Loai Akram Eid MD [6], Marc Fila MD, PhD [7], Michaela Gessner MD [8], Ibrahim Gokce MD [9], Laura Massella MD [10], Antonio Mastrangelo MD [11], Monika Miklaszewska MD, PhD [12], Larisa Prikhodina MD, PhD [13], Bruno Ranchin MD [14], Nadejda Ranguelov MD [15], Rina Rus MD [16], Lale Sever MD [17], Julia Thumfart MD [18], Lutz Thorsten Weber MD [1], Elke Wuehl MD [19], Alev Yilmaz [20], Jörg Dötsch MD [1], Franz Schaefer MD [21], Max Christoph Liebau MD [1,22]

Affiliations:

[1] Department of Pediatrics, University Hospital Cologne and University of Cologne, Faculty of Medicine, Cologne, Germany;

[2] Department of Pediatric Nephrology, Şişli Etfal Training and Research Hospital, İstanbul, Turkey;

[3] Department of Pediatrics II, University Hospital Essen, Essen, Germany;

[4] Department of Pediatric Nephrology, Erciyes University, Faculty of Medicine, Kayseri, Turkey;

[5] Department of Pediatrics, Division of Pediatric Nephrology, Hacettepe University Faculty of Medicine, Ankara, Turkey;

[6] Department of Pediatric Nephrology, Dubai Kidney Center of Excellence, Dubai Hospital, Dubai, United Arab Emirates;

[7] Pediatric Nephrology Unit, CHU Arnaud de Villeneuve-Université de Montpellier, Montpellier, France;

[8] Department of General Pediatrics and Hematology/ Oncology, Children's University Hospital Tuebingen, Germany;

[9] Research and Training Hospital, Division of Pediatric Nephrology, Marmara University, Istanbul, Turkey;

[10] Division of Nephrology, Department of Pediatric Subspecialties, Bambino Gesù Children's Hospital – IRCCS, Rome, Italy;

[11] Pediatric Nephrology, Dialysis and Transplant Unit, Fondazione IRCCS Cà Granda, Ospedale Maggiore Policlinico, Milan, Italy;

[12] Department of Pediatric Nephrology and Hypertension, Faculty of Medicine, Jagiellonian University Medical College, Krakow, Poland;

[13] Department of Inherited and Acquired Kidney Diseases, Research Clinical Institute for Pediatrics n.a. acad. Y. E. Veltishev, Pirogov Russian National Research Medical University, Moscow, Russia;

[14] Pediatric Nephrology Unit, Hôpital Femme Mère Enfant, Hospices Civils de Lyon, Centre de référence maladies rénales rares, Bron, France;

[15] Department of Pediatrics, Université Catholique de Louvain Medical School, Saint-Luc Academic Hospital, Brussels, Belgium;

[16] Division of Nephrology, University Children's Hospital Ljubljana, Ljubljana, Slovenia;

[17] Department of Pediatric Nephrology, Cerrahpaşa School of Medicine, Istanbul University Cerrahpasa, Istanbul, Turkey;

[18] Department of Pediatric Gastroenterology, Nephrology and Metabolic Diseases, Charité – Universitätsmedizin Berlin, Berlin, Germany;

[19] Division of Pediatric Nephrology, Center for Pediatrics and Adolescent Medicine, University of Heidelberg, Heidelberg, Germany;

[20] Pediatric Nephrology Department, Istanbul University Istanbul Medical Faculty, 34662, Istanbul, Turkey;

[21] Division of Pediatric Nephrology, Heidelberg University Center for Pediatrics and Adolescent Medicine, Heidelberg, Germany.

[22] Center for Molecular Medicine, University Hospital Cologne and University of Cologne, Faculty of Medicine, Cologne, Germany;

*these authors contributed equally

Supplementary Figures

Supplementary Figure S1: Flowchart of data inclusion. w/o, without.

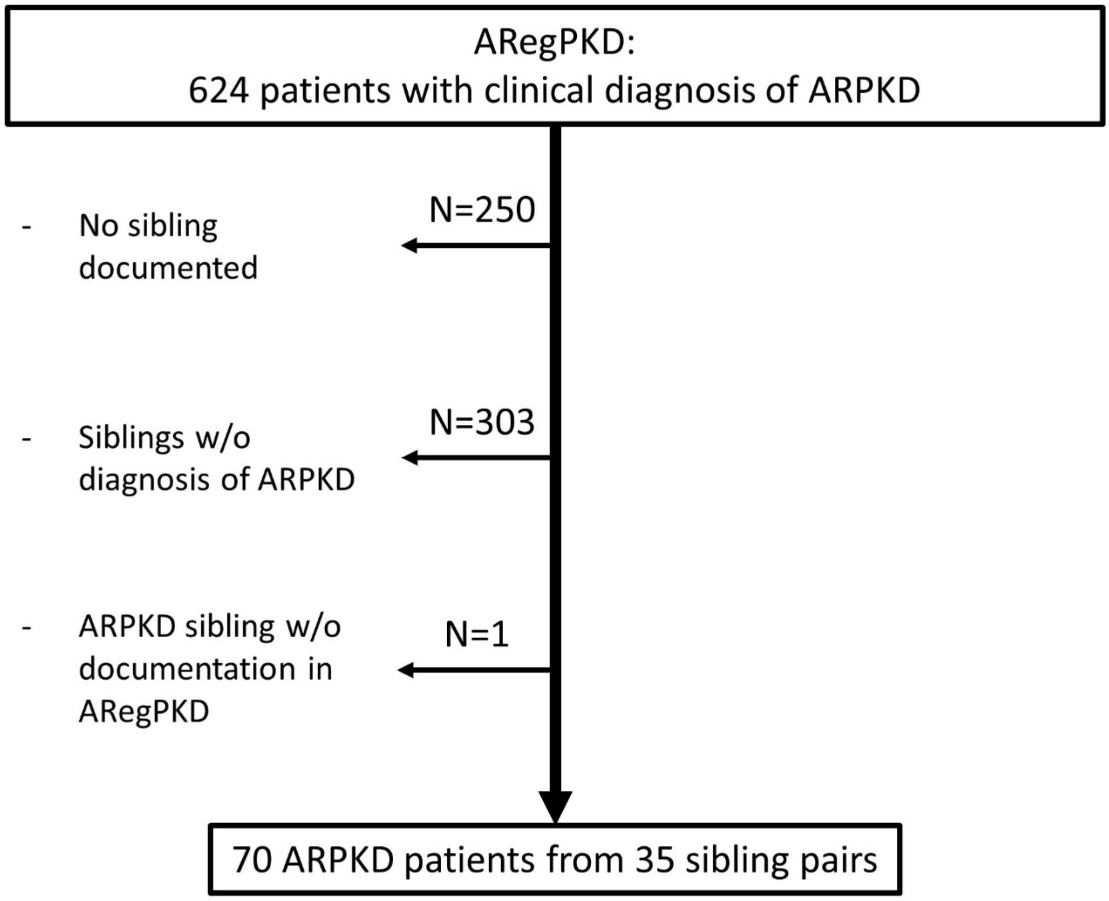

Supplementary Figure S2: First documented visit before and after 2005

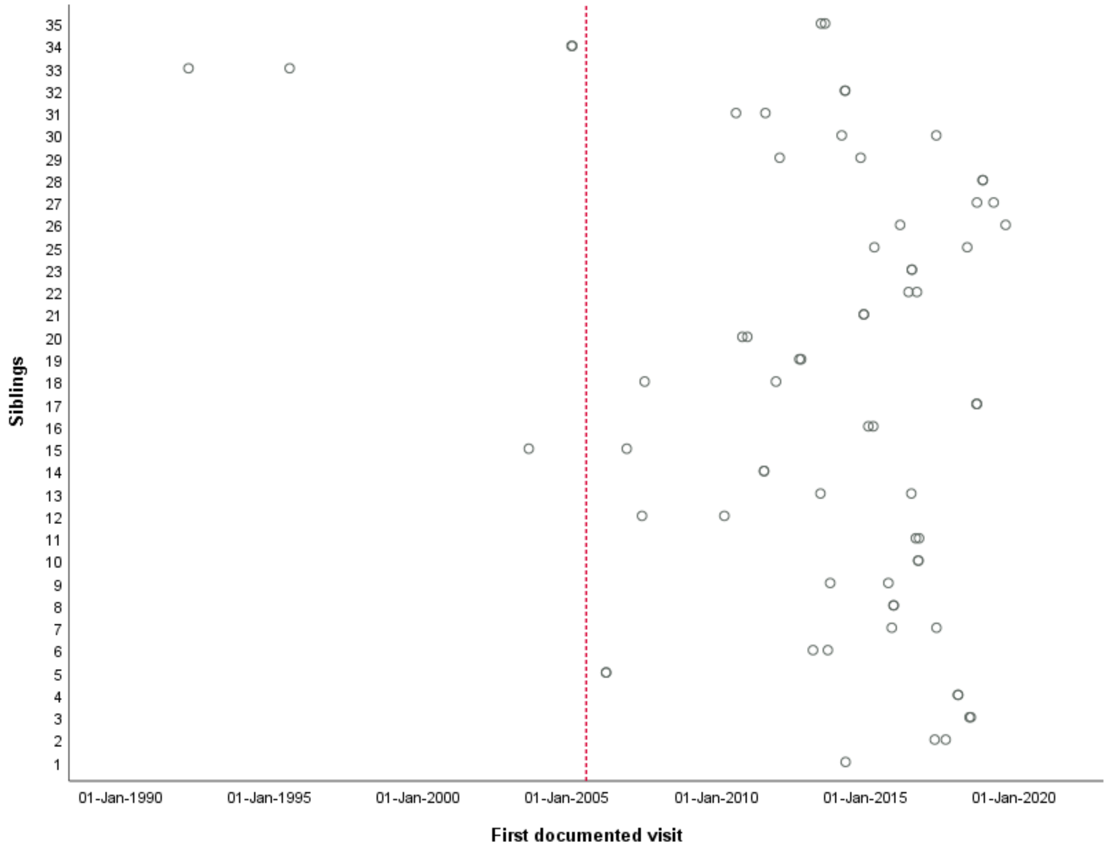

Supplementary Tables

Supplementary Table S1: Overview of genetic testing and findings in *PKHD1* in 70 patients from 35 sibling pairs with ARPKD. m, male; f, female; y, younger sibling; o, older sibling; -, none/not affected/negative/not performed; PU, prenatal ultrasound; PAF, postnatal accidental finding; PE, preventive examination/ well child visit; FS, family screening; O, others, U, unknown; nd, not done; Trunc, Truncating; Mis, Missense; Syn, Synonymous; P, pathogenic (class 5); LP, likely pathogenic (class 4); VUS, variant of uncertain significance (class 3); C, confirmed; U, unknown; LVH, left ventricular hypertrophy; PH, portal hypertension; SHC, severe hepatic complications

**Family ID**	**Siblings, sex, birth order**	**Number of other unaffected siblings**	**Country of origin**	**Con-sanguinity of parents**	**Year of birth**	**Reason of initial pre-sentation at doctor**	**Year of *PKHD1* test**	***PKHD1* variants**	**Literature reference**	**Functionality/ ACMG class**	**Confir-mation of diagnosis (c, p, u)**	**Functional class**	**Average haPTP (cm; age at ultrasound in years)**	**Ratio of haPTP (haPTP older/ haPTP younger)**	**Antihyper-tensive medication until last visit**	**Signs of LVH until last visit**	**CKD G stage at last compar-able visit (age in years)**	**CKD G stage at last visit (age in years)**	**Liver phenotype until last compar-able visit (age in years)**	**Liver phenotype until last visit (age in years)**
1	f2, y	2	Turkey	No	2014	PU	2014	c.8011C>T; c.51A>G	1; 2	Trunc/P; Splice/LP	C	Null/Null	-	-	yes	yes	-	5 (4.8)	-	SHC (4.8)
	m1, o	2	Turkey	No	2002	PU	2015	c.8011C>T; c.51A>G	1; 2	Trunc/P; Splice/LP	C	Null/Null	-	-	-	-	-	-	-	-
2	f2, y	3	Afghanistan	No	2016	FS	2017	c.2798T>A; c.7237C>T; c.7810C>T	3; 3; 3	Mis/VUS; Mis/VUS; Mis/VUS	U	Mis/Mis	-	-	no	no	-	1(1.2)	-	no PH/SHC (1.2)
	m1, o	3	Afghanistan	No	2005	PAF	2017	c.2798T>A; c.7237C>T; c.7810C>T	3; 3; 3	Mis/VUS; Mis/VUS; Mis/VUS	U	Mis/Mis	-	-	no	-	-	1 (13.1)	-	no PH/SHC (13.1)
3	m2, y	-	Italy	No	2003	FS	U	-	-	-	-	-	-	-	yes	no	-	5 (15.4)	-	PH (15.4)
	f1, o	-	Italy	No	1999	PAF	U	-	-	-	-	-	-	-	yes	-	-	5 (18.9)	-	PH (18.9)
4	f2, y	-	Italy	No	2000	PAF	U	-	-	-	-	-	6.6 (18.1)	1.03	no	-	1 (18.1)	1 (18.1)	PH (18.1)	PH (18.1)
	f1, o	-	Italy	No	1999	FS	U	-	-	-	-	-	6.8 (19.9)		no	-	1 (19.0)	1 (19.0)	PH (19.0)	PH (19.0)
5	f2, same age	1	unknown	No	2005	PU	Nd	-	-	-	-	-	11.2 (6.5)	0.56	no	-	4 (11.4)	4 (11.4)	PH (11.4)	PH (11.4)
	f1, same age	1	unknown	No	2005	PU	Nd	-	-	-	-	-	6.3 (6.2)		no	no	4 (11.4)	4 (11.4)	no PH/SHC (11.4)-	no PH/SHC (11.4)
6	f2, y	-	France	No	2001	FS	2013	c.5498C>T; c.2141-9G>A	4; 3	Mis/LP; Splice/LP	C	Others	8.9 (18.0)	0.91	no	-	1 (18.0)	1 (18.0)	no PH/SHC (18.0)	no PH/SHC (18.0)
	f1, o	-	France	No	1999	O	2013	c.5498C>T; c.2141-9G>A	4; 3	Mis/LP; Splice/LP	C	Others	8.1 (17.9)		no	-	1 (18.8)	1 (19.7)	no PH/SHC (18.8)	no PH(SHC (19.7)
	f2, y	2	Algeria	Yes	2016	FS	2017	c.4870C>T; c.4870C>T	5; 5	Mis/P; Mis/P	C	Mis/Mis	-	-	no	-	-	1 (2.2)	-	PH (2.2)

7	m1, o	2	Algeria	Yes	2011	O	2011	c.4870C>T; c.4870C>T	5; 5	Mis/P; Mis/P	C	Mis/Mis	-	-	no	-	-	1 (8.0)	-	no PH/SHC (8.0)
8	m2, y	1	Algeria	No	2001	FS	2005	c.4738C>T; c.4913T>C	6; 3	Mis/VUS; Mis/VUS	U	Mis/Mis	-	-	yes	-	-	1 (18.5)	-	PH (18.5)
	f1, o	1	Algeria	No	1994	O	2005	c.4738C>T; c.4913T>C	6; 3	Mis/VUS; Mis/VUS	U	Mis/Mis	-	-	no	-	-	5 (22.5)	-	PH (22.5)
9	f2, y	1	Portugal	No	2001	PU	2004	c.9718C>T; c.5381-9T>G	7; 4	Trunc/P; Splice/LP	C	Others	-	-	yes	-	2 (17.9)	2 (17.9)	no PH/SHC (17.9)	no PH/SHC (17.9)
	m1, o	1	Portugal	No	1996	O	2004	c.9718C>T; c.5381-9T>G	7; 4	Trunc/P; Splice/LP	C	Others	-	-	yes	-	3 (17.6)	3 (22.0)	no PH/SHC (17.6)	no PH/SHC (22.0)
10	m2, y	1	Algeria	No	2008	O	2016	c.5426G>A; c.10444C>T	8; 7	Mis/LP; Mis/P	C	Mis/Mis	-	-	no	-	-	1 (8.1)	-	SHC (8.1)
	f1, o	1	Algeria	No	1999	U	2016	c.5426G>A; c.10444C>T	8; 7	Mis/LP; Mis/P	C	Mis/Mis	-	-	no	-	-	5 (17.1)	-	SHC (17.1)
11	m2, y	-	Turkey	-	2010	U	Nd	-	-	-	-	-	-	-	yes	-	-	3 (6.4)	-	PH (6.4)
	f1, o	-	Turkey	-	1998	U	Nd	-	-	-	-	-	-	-	yes	-	-	-	-	
12	f2, y	1	Germany	No	2009	PE	Nd	-	-	-	-	-	-	-	yes	yes	3 (9.8)	3 (9.8)	no PH/SHC (9.8)	no PH/SHC (9.8)
	m1, o	1	Germany	No	2007	PE	2017	c.664A>C; c.11398+1G>A	9; 10	Mis/LP; Splice/P	C	Null/Mis	-	-	yes	yes	5 (9.6)	5 (12.5)	PH (9.6)	PH (12.5)
13	m2, y	-	Germany	No	2016	PU	Nd	-	-	-	-	-	-	-	yes	no	3 (3.2)	3 (3.2)	no PH/SHC (3.2)	no PH/SHC (3.2)
	f1, o	-	Germany	No	2013	PAF	Nd	-	-	-	-	-	-	-	yes	-	4 (3.7)	4 (6.5)	PH (3.7)	PH (6.5)
14	f2, same age	-	unknown	No	2010	PAF	2012	c.2171C>G; c.8206T>G	11; 12	Mis/LP; Mis/LP	C	Mis/Mis	10.2 (2.0)	0.93	yes	no	1 (6.0)	1 (6.0)	PH (6.0)	PH (6.0)
	m1, same age	-	unknown	No	2010	FS	2012	c.2171C>G; c.8206T>G	11; 12	Mis/LP; Mis/LP	C	Mis/Mis	10.9 (2.0)		yes	no	1 (6.0)	1(6.0)	PH (6.0)	PH (6.0)
15	m2, y	2	Hungary	-	1996	U	2005	-	-	-	U	-	7.3 (17.5)	0.9	yes	no	1 (19.8)	1 (19.8)	SHC (19.8)	SHC (19.8)
	f1, o	2	Hungary	-	1994	PAF	U	-	-	-	-	-	6.6 (16.9)		yes	no	1 (20.8)	1 (21.8)	SHC (20.8)	SHC (21.8)
16	m2, y	-	Germany	No	2005	PE	2014	c.6992T>A; c.10444C>T	1; 7	Mis/P; Mis/P	C	Mis/Mis	6.2 (13.9)	1.05	no	-	1 (13.9)	1 (13.9)	no PH/SHC (13.9)	no PH/SHC (13.9)
	f1, o	-	Germany	No	2002	O	2014	c.6992T>A; c.10444C>T	1; 7	Mis/P; Mis/P	C	Mis/Mis	6.5 (14.2)		yes	-	1 (13.6)	1 (16.7)	PH (13.6)	PH (16.7)
17	f2, y	-	Azerbaijan	No	2017	FS	Nd	-	-	-	-	-	-	-	yes	no	-	3 (1.2)	-	no PH/SHC (1.2)
	f1, o	-	Azerbaijan	No	2013	PAF	Nd	-	-	-	-	-	-	-	yes	no	-	2 (5.2)	-	SHC (5.2)
18	f2, y	-	Turkey	No	2009	FS	2018	c.4870C>T; c.107C>T	5; 1	Mis/P; Mis/P	C	Mis/Mis	9.6 (6.9)	1.11	yes	-	3 (7.8)	3 (7.9)	no PH/SHC (7.8)	no PH/SHC (7.9)
	f1, o	-	Turkey	No	2006	PAF	2018	c.4870C>T; c.107C>T	5; 1	Mis/P; Mis/P	C	Mis/Mis	10.7 (6.8)		yes	-	2 (8.1)	2 (10.9)	no PH/SHC (8.1)	PH (10.9)
	m2, y	u	Turkey	Yes	2008	PE	Nd	-	-	-	-	-	-	-	yes	-	-	3 (6.5)	-	PH (6.5)

19	m1, o	u	Turkey	Yes	1999	O	Nd	-	-	-	-	-	-	-	no	-	-	1 (18.6)	-	PH (18.6)
20	m2, y	-	Italy	No	2006	O	2013	c.4292G>A; c.4292G>A	13; 13	Mis/P; Mis/P	C	Mis/Mis	-	-	no	no	1 (12.3)	1 (12.3)	PH (12.3)	PH (12.3)
	f1, o	-	Italy	No	2004	FS	2013	c.4292G>A; c.4292G>A	13; 13	Mis/P; Mis/P	C	Mis/Mis	-	-	yes	no	1 (12.4)	1 (14.7)	PH (12.4)	PH (14.7)
21	f2, y	1	Turkey	Yes	2003	O	Nd	-	-	-	-	-	6.5 (13.6)	1.1	yes	-	1 (13.6)	1 (13.6)	PH (13.6)	PH (13.6)
	f1, o	1	Turkey	Yes	2001	O	Nd	-	-	-	-	-	7.2 (14.7)		yes	-	1 (13.0)	1 (14.7)	SHC (13.0)	SHC (14.7)
22	m2, y	2	Turkey	No	2016	PU	Nd	-	-	-	-	-	-	-	yes	-	-	4 (0.4)	-	no PH/SHC (0.4)
	m1, o	2	Turkey	No	2015	PU	Nd	-	-	-	-	-	-	-	yes	-	-	5 (2.9)	-	no PH/SHC (2.9)
23	f2, y	-	Egypt	No	2016	PU	Nd	-	-	-	-	-	9.1 (4.0)	1.3	yes	-	2 (4.0)	2 (4.0)	no PH/SHC (4.0)	no PH/SHC (4.0)
	f1, o	-	Egypt	No	2013	PU	Nd	-	-	-	-	-	11.9 (3.1)		yes	-	3 (4.0)	2 (6.6)	no PH/SHC (4.0)	no PH/SHC (6.6)
24	f2, y	-	Togo/Ghana	No	2010	PU	2011	-	-	-	U	-	-	-	-	-	-	-	-	-
	m1, o	-	Togo/Ghana	No	2007	PU	2008	c.3467C>T	14	Mis/P	U	Single variant	-	-	-	-	-	-	-	-
25	m2, y	-	Turkey	-	2014	PU	Nd	-	-	-	-	-	-	-	yes	yes	-	1 (2.2)	-	no PH/SHC (2.2)
	m1, o	-	Turkey	-	2007	O	Nd	-	-	-	-	-	-	-	yes	no	-	1 (11.1)	-	no PH/SHC (11.1)
26	m2, y	-	Turkey	No	2017	FS	Nd	-	-	-	-	-	-	-	no	-	3 (2.3)	3 (2.3)	no PH/SHC (2.3)	no PH/SHC (2.3)
	m1, o	-	Turkey	No	2015	PE	2016	c.10972_10973 delAT;	7	Trunc/P;	U	Single variant	-	-	yes	no	2 (3.3)	2 (3.3)	no PH/SHC (3.3)	no PH/SHC (3.3)
27	f2, y	2	Syria	Yes	2012	O	2019	c.4870C>T; c.4870C>T	5; 5	Mis/P; Mis/P	C	Mis/Mis	-	-	no	-	-	1 (6.6)	-	PH (6.6)
	m1, o	2	Syria	Yes	2010	FS	Nd	-	-	-	-	-	-	-	no	-	-	1 (9.3)	-	no PH/SHC (9.3)
28	m2, y	4	Syria	Yes	2017	FS	2018	c.2397A>G; c.2397A>G	3; 3	Syn/VUS; Syn/VUS	U	Others	-	-	no	-	-	1 (1.9)	-	no PH/SHC (1.9)
	f1, o	4	Syria	Yes	2005	O	2018	c.2397A>G; c.2397A>G	3; 3	Syn/VUS; Syn/VUS	U	Others	-	-	no	-	-	4(13.9)	-	PH (13.9)
29	m2, y	-	Poland	No	2014	FS	Nd	-	-	-	-	-	9.2 (4.6)	1.11	yes	-	1 (4.6)	1(4.6)	PH (4.6)	PH (4.6)
	m1, o	-	Poland	No	2011	O	2016	c.8114delG;	12	Trunc/P;	U	Single variant	10.2 (4.8)		yes	-	1 (4.8)	1 (7.8)	PH (4.8)	PH (7.8)
30	m2, y	-	Poland	No	2017	PU	Nd	-	-	-	-	-	-	-	yes	-	4 (1.3)	4 (2.3)	no PH/SHC (1.3)	no PH/SHC (2.3)
	f1, o	-	Poland	No	2013	O	U	-	-	-	-	-	-	-	yes	-	2 (1.5)	3 (5.6)	no PH/SHC (1.5)	no PH/SHC (5.6)

31	f2, y	-	Russia	-	2007	FS	2018	c.2725C>T;<br>c.4870C>T	15;<br>5	Trunc/P;<br>Mis/P	C	Null/Mis	8.9 (6.5)	1.13	yes	-	2 (6.5)	2 (6.5)	no<br>PH/SHC<br>(6.5)	no<br>PH/SHC<br>(6.5)
	m1, o	-	Russia	-	2003	PAF	2018	c.2725C>T;<br>c.4870C>T	15;<br>5	Trunc/P;<br>Mis/P	C	Null/Mis	10.1 (7.2)		yes	no	2 (7.2)	2 (11.0)	no<br>PH/SHC<br>(7.2)	no<br>PH/SHC<br>(11.0)
32	f2, same<br>age	-	unknown	No	2014	PU	2014	c.5498C>T;<br>c.(7380_7525)_<br>(7526_7860)del	4;<br>3	Mis/LP;<br>Exon del/P	C	Null/Mis	7.8 (5.4)	1.3	no	no	1 (4.2)	1 (5.4)	no<br>PH/SHC<br>(4.2)	no<br>PH/SHC<br>(5.4)
	f1, same<br>age	-	unknown	No	2014	PU	2014	c.5498C>T;<br>c.(7380_7525)_<br>(7526_7860)del	4;<br>3	Mis/LP;<br>Exon del/P	C	Null/Mis	10.1 (5.3)		yes	no	1 (4.2)	1 (5.3)	no<br>PH/SHC<br>(4.2)	no<br>PH/SHC<br>(5.3)
33	m2, y	4	unknown	No	1995	PU	U	-	-	-	-	-	14.9 (4.2)	0.63	yes	yes	5 (19.0)	5 (23.3)	no<br>PH/SHC<br>(19.0)	PH (23.3)
	m1, o	4	unknown	No	1991	O	Nd	-	-	-	-	-	9.5 (4.2)		no	-	2 (19.4)	2 (26.8)	PH (19.4)	PH (26.8)
34	m2, y	-	unknown	No	2005	O	2005	c.107C>T;<br>c.10226A>T	1;<br>6	Mis/P;<br>Mis/P	C	Mis/Mis	12.2 (10.5)	0.62	yes	yes	1 (13.5)	1 (13.5)	SHC<br>(13.5)	SHC<br>(13.5)
	f1, o	-	unknown	No	2002	PU	2005	c.107C>T;<br>c.10226A>T	1;<br>6	Mis/P;<br>Mis/P	C	Mis/Mis	7.6 (10.2)		yes	yes	1 (12.9)	1(15.8)	SHC<br>(12.9)	(SHC<br>15.8)
35	f2, y	-	Slovenia	No	2012	O	2014	c.8636A>G;<br>c.11219C>T	-;<br>-	Mis/VUS;<br>Mis/VUS	U	Mis/Mis	-	-	yes	-	-	1 (7.4)	-	PH (7.4)
	m1, o	-	Slovenia	No	2010	FS	2014	c.8636A>G;<br>c.11219C>T	-;<br>-	Mis/VUS;<br>Mis/VUS	U	Mis/Mis	-	-	yes	-	-	3 (8.9)	-	PH (8.9)

Supplementary Table S2: Perinatal data of patients requiring kidney replacement therapy (KRT) and their corresponding siblings. m, male; f, female; +, yes; -, no; u, unknown; n.a., not available; CLKTx, combined liver and kidney transplantation; KTx, kidney transplantation.

**Siblings, sex, birth order**	**Perinatal assisted breathing and/or ventilation**	**Prematurity (gestational age at birth, weeks)**	**Birth weight (g)**	**APGAR at 1/5/10 min**	**Age at death or KRT or CKD G stage at last visit (years)**
1 Sib1m1, older	+	+ (34+6)	2600	6/7/9	Death (0.07)
1 Sib1f2, younger	+	+ (31+1)	2010	6/6/8	+ CLKTx (3.1)
3 Sib3f1, older	-	- (38+0)	3040	n.a.	+ KTx (6.8)
3 Sib3m2, younger	-	- (37+0)	u	n.a.	+ KTx (7.4)
8 Sib8f1, older	-	- (38+0)	3000	n.a.	+KTx (21.8)
8 Sib8m2, younger	-	u	u	n.a.	CKD G1 (18.5)
10 Sib10f1, older	-	u	u	n.a.	+ Dialysis (9.3)
10 Sib10m2, younger	-	u	u	n.a.	CKD G1 (8.1)
12 Sib12m1, older	-	- (41+4)	3450	9/10/10	+ KTx (7.6)
12 Sib12f2, younger	-	- (39+4)	2555	9/10/n.a.	CKD G3 (9.8)
22 Sib22m1, older	-	- (39+6)	3500	n.a./10/10	+ Dialysis (0.9)
22 Sib22m2, younger	+	+ (30+2)	1800	n.a.	CKD G4 (0.4)
33 Sib33m1, older	-	- (37+0)	3220	10/10/10	CKD G2 (26.8)
33 Sib33m2, younger	u	- (39+0)	3980	8/8/n.a.	+ KTx (11.9)

Modified STROBE Statement—checklist of items that should be included in reports of observational studies (Cohort/Cross-sectional and case-control studies)

	Item No	Recommendation	
**Title and abstract**	1	(*a*) Indicate the study's design with a commonly used term in the title or the abstract	See abstract on page 3 of submitted manuscript word file
		(*b*) Provide in the abstract an informative and balanced summary of what was done and what was found	
**Introduction**			
Background/rationale	2	Explain the scientific background and rationale for the investigation being reported	See pages 4 and 5 of submitted manuscript word file
Objectives	3	State specific objectives, including any prespecified hypotheses	See pages 4 and 5 of submitted manuscript word file
**Methods**			
Study design	4	Present key elements of study design early in the paper	See page 6 of submitted manuscript word file
Setting	5	Describe the setting, locations, and relevant dates, including periods of recruitment, exposure, follow-up, and data collection	See pages 6-8 of submitted manuscript word file, Table 1, Supplemental Table 1 and Supplemental Figure S1
Participants	6	(*a*) *Cohort study*—Give the eligibility criteria, and the sources and methods of selection of participants. Describe methods of follow-up	See page 6 of submitted manuscript word file and cited references
Variables	7	Clearly define all outcomes, exposures, predictors, potential confounders, and effect modifiers. Give diagnostic criteria, if applicable	See pages 6-8 of submitted manuscript word file
Data sources/ measurement	8*	For each variable of interest, give sources of data and details of methods of assessment (measurement).	See page 6-7 of submitted manuscript word file

Bias	9	Describe any efforts to address potential sources of bias	See page 6 of submitted manuscript word file
Study size	10	Explain how the study size was arrived at (if applicable)	Does not apply
Quantitative variables	11	Explain how quantitative variables were handled in the analyses. If applicable, describe which groupings were chosen and why	See pages 6-7 of submitted manuscript word file and description of subgroups in results section on pages 8-13
Statistical methods	12	(*a*) Describe all statistical methods, including those used to control for confounding	See page 7 of submitted manuscript
		(*b*) Describe any methods used to examine subgroups and interactions	See pages 6-8 and 8-13 of submitted manuscript
		(*c*) Explain how missing data were addressed	See page 7 of submitted manuscript
		(*d*) *Cohort study*—If applicable, explain how loss to follow-up was addressed	Does not apply
		(*e*) Describe any sensitivity analyses	Does not apply
**Results**			
Participants	13*	(a) Report numbers of individuals at each stage of study—eg numbers potentially eligible, examined for eligibility, confirmed eligible, included in the study, completing follow-up, and analyzed	See page 8 of submitted manuscript word file table 1, supplementary table 1 and all figures
		(c) **Use of a flow diagram**	See Suppl Figure 1
Descriptive data	14*	(a) Give characteristics of study participants (eg demographic, clinical, social) and information on exposures and potential confounders	See table 1, and suppl tables 1 and 2
		(b) Indicate number of participants with missing data for each variable of interest	See page 7 for description of definition of informative cases and all tables
		(c) *Cohort study*—Summarise follow-up time (eg, average and total amount)	See table 1

Outcome data	15*	*Cohort study*—Report numbers of outcome events or summary measures over time	See Figures 2 and 3
Main results	16	(*a*) Give unadjusted estimates and, if applicable, confounder-adjusted estimates and their precision (eg, 95% confidence interval). Make clear which confounders were adjusted for and why they were included	See Figures 1-3
Other analyses	17	Report other analyses done—eg analyses of subgroups and interactions, and sensitivity analyses	See pages 8-13 and all figures
**Discussion**			
Key results	18	Summarise key results with reference to study objectives	See pages 14-16 of submitted manuscript word file
Limitations	19	Discuss limitations of the study, taking into account sources of potential bias or imprecision. Discuss both direction and magnitude of any potential bias	See page 16 of submitted manuscript word file
Interpretation	20	Give a cautious overall interpretation of results considering objectives, limitations, multiplicity of analyses, results from similar studies, and other relevant evidence	See page 14-16 of submitted manuscript
Generalisability	21	Discuss the generalisability (external validity) of the study results	See pages 15&16 of submitted manuscript word file

*Give information separately for cases and controls in case-control studies and, if applicable, for exposed and unexposed groups in cohort and cross-sectional studies.

Note: An Explanation and Elaboration article discusses each checklist item and gives methodological background and published examples of transparent reporting. The STROBE checklist is best used in conjunction with this article (freely available on the Web sites of PLoS Medicine at http://www.plosmedicine.org/, Annals of Internal Medicine at http://www.annals.org/, and Epidemiology at http://www.epidem.com/). Information on the STROBE Initiative is available at www.strobe-statement.org.
